# MiR-21-5p regulates the dynamic of mitochondria network and rejuvenates the senile phenotype of bone marrow stromal cells (BMSCs) isolated from osteoporotic SAM/P6 mice

**DOI:** 10.1186/s13287-023-03271-1

**Published:** 2023-03-29

**Authors:** Mateusz Sikora, Agnieszka Śmieszek, Ariadna Pielok, Krzysztof Marycz

**Affiliations:** 1 Department of Experimental Biology, The Faculty of Biology and Animal Science, University of Environmental and Life Sciences Wroclaw, Norwida 27B St, 50-375 Wrocław, Poland; 2 Department of Medicine and Epidemiology, School of Veterinary Medicine, University of California, One Shields Avenue, Davis, CA 95616-8739 USA; 3 International Institute of Translational Medicine, Jesionowa 11 Street, 55-124 Malin, Poland

**Keywords:** miR-21, Senile osteoporosis, Bone regeneration, Mitochondria dynamic, BMSCs, SAM/P6 mice

## Abstract

**Background:**

Progression of senile osteoporosis is associated with deteriorated regenerative potential of bone marrow-derived mesenchymal stem/stromal cells (BMSCs). According to the recent results, the senescent phenotype of osteoporotic cells strongly correlates with impaired regulation of mitochondria dynamics. Moreover, due to the ageing of population and growing osteoporosis incidence, more efficient methods concerning BMSCs rejuvenation are intensely investigated. Recently, miR-21-5p was reported to play a vital role in bone turnover, but its therapeutic mechanisms in progenitor cells delivered from senile osteoporotic patients remain unclear. Therefore, the goal of this paper was to investigate for the first time the regenerative potential of miR-21-5p in the process of mitochondrial network regulation and stemness restoration using the unique model of BMSCs isolated from senile osteoporotic SAM/P6 mice model.

**Methods:**

BMSCs were isolated from healthy BALB/c and osteoporotic SAM/P6 mice. We analysed the impact of miR-21-5p on the expression of crucial markers related to cells’ viability, mitochondria reconstruction and autophagy progression. Further, we established the expression of markers vital for bone homeostasis, as well as defined the composition of extracellular matrix in osteogenic cultures. The regenerative potential of miR-21 in vivo was also investigated using a critical-size cranial defect model by computed microtomography and SEM–EDX imaging.

**Results:**

MiR-21 upregulation improved cells’ viability and drove mitochondria dynamics in osteoporotic BMSCs evidenced by the intensification of fission processes. Simultaneously, miR-21 enhanced the osteogenic differentiation of BMSCs evidenced by increased expression of *Runx-2* but downregulated *Trap*, as well as improved calcification of extracellular matrix. Importantly, the analyses using the critical-size cranial defect model indicated on a greater ratio of newly formed tissue after miR-21 application, as well as upregulated content of calcium and phosphorus within the defect site.

**Conclusions:**

Our results demonstrate that miR-21-5p regulates the fission and fusion processes of mitochondria and facilitates the stemness restoration of senile osteoporotic BMSCs. At the same time, it enhances the expression of RUNX-2, while reduces TRAP accumulation in the cells with deteriorated phenotype. Therefore, miR-21-5p may bring a novel molecular strategy for senile osteoporosis diagnostics and treatment.

**Supplementary Information:**

The online version contains supplementary material available at 10.1186/s13287-023-03271-1.

## Background

Senile osteoporosis (OP) belongs to the progressive skeletal system diseases, manifested by disproportionate bone loss. Osteoporosis development results from the imbalance between bone resorption and bone formation, leading to gradual losses of bone mass and deteriorated density due to its demineralization [1–3]. As reported by World Health Organization (WHO) and International Osteoporosis Foundation (IOF), more than 200 million women worldwide are affected by this disability. Moreover, OP is diagnosed in more than 75 million people in Europe, Japan and the USA [4]. It is estimated that by 2050 osteoporosis-dependent bone fractures will increase to 310% in men and 240% in women when compared to the '90 s [5]. Furthermore, patients suffering from osteoporosis-related fractures are life-threatening, since they usually become bedridden and require constant medical health care [6]. This alarming data clearly indicate that OP is a progressing epidemy affecting people worldwide and require the development of intelligent pharmaceutical strategies to reverse this unfavourable trend.

Recently, bone marrow-derived mesenchymal stem/stromal cells (BMSCs) have been proposed to have clinical potential for osteoporosis-related bone fractures regeneration due to their unique physiological properties [7, 8]. Numerous studies showed that BMSCs compared to the other stem cells populations exhibit a higher ability to stimulate and enhance bone regeneration due to their paracrine activity [9, 10]. BMSCs were also identified as osteogenic stem/progenitor cells, demonstrating high osteoblast differentiation potential, hence their therapeutic potential is mainly associated with bone tissue regeneration [9, 11]. However, MSCs (mesenchymal stem cells) are losing their regenerative ability with age, and MSCs derived from elderly patients have limited regenerative potential [12–14]. Importantly, we have previously shown that BMSCs obtained from senile accelerated osteoporotic SAM/P6 mice were characterised by poor regenerative potential accompanied with stemness deterioration, which contributes to osteoporosis progression and might become a pharmaceutical target to prevent OP development [8]. Further, we have indicated an upregulated accumulation of reactive oxygen species (ROS) and a greater ratio of mitochondria with depolarized membrane potential within osteoporotic BMSCs that indicate on deteriorated cytophysiological processes [8]. Furthermore, osteoporotic BMSCs presented inflammatory features, e.g. upregulation of TRAP expression, which undeniably links osteoporosis state with ongoing inflammation [8, 15–17]. Therefore, searching for an effective therapeutic solution that would target BMSCs rejuvenation and stemness restoration seems to be fully justified.

Recently, microRNAs (miRNAs) have been shown to play an essential regulatory role in bone cells growth, differentiation potential, metabolic activity, regulation of multipotency, as well as mitochondrial dynamics [2, 18]. MiR-21-5p (miR-21) is differentially expressed in many diseases, leading to pathological changes. The miR-21-5p level was reported to be reduced in serum and bone tissue of osteoporotic patients, hence may serve as a potential diagnostic biomarker of OP in liquid biopsies [19]. However, it has never been elucidated if the upregulation of miR-21 could affect the stemness restoration by the regulation of the mitochondria network in bone tissue milieu of senile osteoporotic patients. Nonetheless, our previous studies provide evidence that regulation of miR-21 expression may contribute to proper bone homeostasis, e.g. by modulating the expression of markers that regulate osteoblasts differentiation and drive their interplay with osteoclasts [2, 20, 21]. Concluding, we hypothesised that miR-21-5p may serve as an important factor with the evident capability to recover the deteriorated stemness of BMSCs in senile osteoporotic patients, e.g. by the regulation of mitochondria network dynamic that affects the cells’ capability for bone homeostasis maintenance.

In this study, we assess whether the application of miR-21-5p rescues bone marrow mesenchymal stem/stromal cells (BMSC_SAM/P6_) from senile phenotype and defective osteogenesis. The BMSC_SAM/P6_ and BMSC_SAM/P6+miR-21_ were compared to healthy BMSCs isolated from wild-type BALB/c mice (BMSC_BALB/c_). We have analysed for the first time the impact of miR-21 on the regulation of mitochondrial network dynamic evidenced by differences in fusion and fission processes, as well as restoration of cells’ stemness, including the potential for osteogenic differentiation. Therefore, we also focused on the miR-21-5p role in BMSCs metabolism and autophagy. Moreover, we have examined the cells’ capability to differentiate into bone tissue by assessing the expression of markers associated with bone remodelling, both on RNA and protein levels. Finally, we have evaluated the miR-21 impact on bone regeneration in vivo, using a critical-size cranial defect model (CSD) accompanied by computed microtomography (µ-CT) and SEM–EDX analyses. Given the obtained results, we speculate that miR-21-5p might become a potential therapeutic molecule for treating osteoporotic-related fractures by senile BMSCs rejuvenation. Our studies provide evidence that miR-21-5p has a pro-osteogenic function restoring the differentiation potential of progenitor cells with deteriorated metabolism. These results indicate that miR-21-5p is a promising target which can be modulated in order to regulate BMSCs metabolism and mitochondria network dynamic, thus, to restore the loss regenerative potential of BMSC for treatment of senile osteoporosis.

## Methods

### Model of senescence-accelerated mice prone 6

SAM/P6 mice strain (senescence-accelerated mice prone 6) was used as an animal model of senile osteoporosis. The strain belongs to SAM group of inbred mouse strains that are used as animal models of senescence acceleration, as well as age-related disorders. The SAM/P6 strain was obtained by Dr. Takeda during brother-sister mating of AKR/J mice in Kyoto University. In the age of 4 months, SAM/P6 mice presents the full spectrum of senile osteoporosis symptoms: low global bone density, deteriorated osteogenesis of bone marrow, deficits in endocortical mineralizing surface, reduction of BMD index and BV/TV index, as well as reduced calcium and phosphorus level. In addition, SAM/P6 mice are characterised by age-dependent inhibition of osteoblastogenesis and osteoclastogenesis but enhanced adipogenesis, which results in early disturbances of bone homeostasis [22–24].

### The procedure of isolation and propagation of bone marrow-delivered mesenchymal stem/stromal cells

Long bones of mice lower limbs (femurs) were isolated in order to collect bone marrow-delivered mesenchymal stem/stromal cells (BMSCs). In order to collect the bones, the mice had to be sacrificed. Before the sacrifice procedure, the animals were given an aesthetic (mixture of xylazine—15 mg/kg and ketamine—50 mg/kg) in order to minimalize the stress and suffering of animals. 15 min after administration of anaesthetics, animals were euthanized by the disruption of the spinal cord. The procedure was performed by a person qualified to kill animals. The death of each animal was confirmed by a veterinarian. The cells were isolated from two mice strains: healthy BALB/c mice (n = 15 mice, 4 months old) and senescence-accelerated mice prone 6—SAM/P6 (n = 15 mice, 4 months old). Collected bones (two bones per mouse) were washed two times using Hank’s Balanced Salt Solution (HBSS) with 1% addition of P/S (penicillin & streptomycin). The bone marrows were isolated by flushing it from medullary canals by the use of insulin syringes U-40 (29G X 1/2′′ needle) filled with HBSS and the samples were pooled together within the strains. After double centrifugation (300×*g*, 4 min), they were counted using Muse® Count & Viability Kit (Merck®; cat. no.: MCH100102, Poznan, Poland) accordingly to manufacturers’ instructions. Then, the isolated cells were inoculated on the 24-well dishes (800 000 cell/well) in 500 µL of Ham’s F-12 Nutrient Mixture (F-12) supplemented with 15% of foetal bovine serum (FBS) and 1% of P/S. The cells were maintained in CO_2_ incubator (5% CO_2_, 37 °C and 95% humidity). The culture media were removed and replaced with fresh media after 24 h of propagation in order to eliminate non-adherent hematopoietic cell lineage [25–27]. During cells propagation, the media were changed every 3–4 days.

### Transfection with miR-21 Mimic

For the purpose of transfection, BMSCs were trypsinized at least once and propagated on the 24-well plates until reaching approximately 80% of confluency. Then, BMSCs isolated from SAM/P6 mice were transfected using MISSION® miRNA miR-21 Mimic (HMI0371, Sigma-Aldrich, Munich, Germany) and Lipofectamine 3000 Transfection Kit (L3000-008, Invitrogen, Thermo Fisher Scientific, Warsaw, Poland). The procedure of transfection was conducted accordingly to manufacturers protocol. The Lipofectamine 3000 Reagent and MISSION® miR-21 miRNA Mimic were prepared in OptiMEM medium (31985-070, Gibco, Life Technologies Corporation, USA) and mixed as described in producer’s instructions. The transfection reagent was added to the cultures in the dilution 1:10. MiR-21 was used at the final concentration of 50 nM. The procedure of cells’ transfection has been carried out for 72 h. BMSCs isolated from SAM/P6 mice that were not transfected with miR-21-5p, as well as BMSCs isolated from healthy BALB/c mice served as references during the experiment. After transfection, the cells were collected or maintained for osteogenic differentiation.

### Cytometric evaluation of proteins related to bone homeostasis

In order to analyse the presence of proteins related to bone homeostasis, cytometric evaluation was performed. After the bone marrow isolation, the cells were inoculated on the 24-well plates. After 24 h, the cells were washed with HBSS and immediately transfected with miR-21 Mimic for 72 h, as described previously. Further, the cells were trypsinized, centrifuged (1300 RPM, 5 min, 4 °C), washed in PBS (Phosphate Buffered Saline) with 2% addition of FBS and centrifuged for the second time (1300 RPM, 5 min, 4 °C). Finally, the cells were washed with PBS without FBS and centrifuged for the third time (1300 RPM, 5 min, 4 °C). In order to lyse the remained erythrocytes, 1 mL of NH4Cl was added to the samples and centrifuged (1300 RPM, 5 min, 4 °C). Then, the samples were incubated with NH4Cl for 15 min and centrifuged (1300 RPM, 5 min, 4 °C). Next, 100 µL of Fix & Perm Medium A (GAS001, Life Technologies Corporation, USA) was added to the cells and incubated for 15 min. Then, the cells were washed with 3 mL of PBS with 5% addition of FBS and centrifuged (350×*g*, 5 min). After that, the cells were incubated in the dark for 30 min with Fix & Perm Medium B (GAS002, Life Technologies Corporation, USA) with the addition of anti-RUNX-2 (M-70) antibody produced in rabbit (sc-10758, Santa Cruz Biotechnology) in the dilution 1:50. The cells were washed in 3 mL of PBS with 5% addition of FBS and centrifuged (350×*g*, 5 min). The secondary antibody—Anti-rabbit Atto-647 produced in goat (ab150079, Abcam)—was diluted in the PBS at the concentration 1:100 and incubated with the samples for 30 min in the dark. The samples were washed in 3 mL of PBS without FBS and centrifuged (350×*g*, 5 min). The cells were resuspended in 500 µL of fresh PBS and proceeded for cytometric evaluation. The samples were analysed using two-laser FACS Lyric Flow Cytometer (Becton Dickinson Polska, Sp. z o.o., Warsaw, Poland) with FASC Suite software. In each sample, 1000 of cells were evaluated. The results were visualised and analysed using FCS Express™ Software (version 7.08.0018, De Novo Software, Pasadena, CA, USA).

### Osteogenesis induced in BMSCs

In order to induce the osteogenic differentiation of BMSCs, the cultures were propagated in the osteogenic medium prepared as described previously [20, 25]: MEM-α medium (Minimum Essential Medium Eagle—Alpha Modification) was supplemented with ascorbic acid (50 µg/mL), β glycerol phosphate disodium salt hydrate (10 nM) and 15% addition of FBS. The osteogenesis was performed for 10 days, and the media were changed twice a week. After differentiation, the cells were collected for analysis.

### Evaluation of extracellular matrix composition

After osteogenic differentiation, the cells were fixed with 4% PFA (paraformaldehyde) for 15 min at room temperature. To visualise the calcium deposits, the specimens were stained with Alizarin Red for 20 min at room temperature as described previously [20, 28]. Then, the specimens were washed three times with distilled water and observed under Axio Observer A1 inverted microscope (3832000970, Zeiss, Oberkochen, Germany). The photographs were taken by Canon PowerShot digital camera (Woodhatch, UK) under 100 × magnification. The resolution of obtained images was: 3648 × 2736 pixels. The differences in staining intensity between the specimens were based on the number of colour pixels. The number of colour pixels was determined in three technical repetitions and using three different thresholds in Pixel Counter plugin (ImageJ Software version 1.52n, Wayne Rasband, National Institutes of Health, USA).

### Immunocytochemical detection of proteins accumulation and cells’ ultrastructure

The immunocytochemical staining technique was used to visualise the cells’ ultrastructure and protein accumulation, as described previously [8] and accordingly to manufacturers’ instructions. Mitochondrial network was stained using Mito Red dye (Sigma-Aldrich, Munich, Germany) at the concentration of 1:1000 in CGM (complete growth medium) for 30 min at 37 °C. The lysosomes were visualised using LysoTracker™ Yellow HCK-123 (Life Technologies Corporation, USA) at the concentration of 1:10,000 in CGM for 30 min at 37 °C. Before subsequent stainings, the cells were fixed with 4% PFA for 15 min and washed three times with HBSS. Afterwards, the specimens were permeabilized using 0.2% PBS-Tween solution with 10% addition of goat serum for 1 h and washed 3 times with HBSS. The cells’ integrity was assessed by nuclei visualisation using Hoechst 33,342 (I34202, Invitrogen, Thermo Fisher Scientific, Warsaw, Poland) dye that was performed by incubation of specimens at 37 °C for 5 min. The reagent was diluted to the concentration 2 µg/mL. Moreover, the actin cytoskeleton was stained using phalloidin solution (49409, Sigma-Aldrich, Munich, Germany) at the concentration 1:800 for 40 min at 37 °C. Then, cells were incubated overnight with primary antibodies at 4 °C. The antibodies used for staining were diluted in HBSS: anti-RUNX-2 antibody (F-2) produced in mice (sc-390351, Santa Cruz Biotechnology, Dallas, Texas, USA) at the concentration 1:50 and anti-TRAP antibody (D-3) mouse monoclonal IgG1 (sc-376875, Santa Cruz Biotechnology, Dallas, Texas, USA) at the concentration 1:50. RUNX-2 and TRAP are initial markers of bone homeostasis and participate in bone turnover processes. Additionally, the specimens were stained with anti-LAMP2 antibody (H4B4) produced in mouse (ab25631, Abcam, Cambridge, UK) at the concentration 1:100 that serves as a marker of selective autophagy; anti-Ki67 antibody produced in rabbit (ab15580, Abcam, Cambridge, UK) at the concentration 1:1000 that is cells’ proliferation marker; anti mTOR antibody (nb100-240) produced in rabbit (Novus Biologicals, Bio-Techne) at the concentration 1:100; and anti-MFN-1 antibody produced in rabbit (orb11040, Biorbyt) at the concentration 1:250 which is a mediator of mitochondria fusion. For the purpose of BMSCs immunophenotyping, the following antibodies were used: anti-CD44 produced in rabbit (hpa005785, Sigma-Aldrich, Munich, Germany) at the concentration 1:1000; anti-CD45 produced in mouse (sc-53047, Santa Cruz Biotechnology, Dallas, Texas, USA) at the concentration 1:100; anti-CD73 produced in mouse (ab54217, Abcam, Cambridge, UK) at the concentration 0.1 µg/100µL; anti-CD90 produced in rabbit (ab92574, Abcam, Cambridge, UK) at the concentration 1:100 and anti-CD105 produced in rabbit (ab107595, Abcam, Cambridge, UK) at the concentration 1:100. Subsequently, the specimens were washed three times with HBSS and incubated with secondary antibodies: IgG—Atto 594 antibody produced in goat (anti-mouse or anti-rabbit) at the concentration 1:100 (Sigma-Aldrich, Munich, Germany) for 1 h at room temperature. The specimens were washed three times with HBSS and stained with DAPI (4’,6-diamino-2-phenolindole) using a mounting medium (FluoroshieldTM with DAPI, Sigma-Aldrich, Munich, Germany). The cells were observed using a confocal microscope and Las X software (11889113, Leica DMi8, Leica Microsystems, KAWA.SKA Sp. z o.o., Zalesie Górne, Poland). The images were captured under 630 × and 1000 × magnification. The microscopic images were obtained by applying maximum intensity projection using Fiji is just ImageJ Software (version 1.52n, Wayne Rasband, National Institutes of Health, USA). The obtained images resolution was 768 × 256 pixels. The differences in staining intensity between the specimens were evaluated based on the number of colour pixels from images captured in three technical repetitions and using three different thresholds (thresholds: 49, 50, 51) in Pixel Counter plugin (ImageJ Software). Additionally, the MicroP Software was used to analyse the mitochondria morphology [29].

### Evaluation of miRNA and mRNA expression in BMSCs cultures

The miRNA and mRNA transcripts levels were analysed using RT-qPCR (reverse transcription quantitative polymerase chain reaction) technique as described previously in detail [8, 28]. The cells were homogenised using 1 mL of Extrazol® (Blirt DNA, Gdańsk, Poland). Then, RNA was isolated using the phenol–chloroform method. The isolated RNA was diluted in molecular grade water (Sigma-Aldrich, Poznan, Poland) and evaluated spectrophotometrically (Epoch, Biotek, Bad Friedrichshall, Germany). Digestion of gDNA was performed using PrecisionDNAse Kit (Primerdesign, BLIRT S.A., Gdańsk, Poland). cDNA was synthesised using Tetro cDNA Synthesis Kit (Bioline Reagents Limited, London, UK) in T100 Thermal Cycler (Bio-Rad, Hercules, CA, USA). For non-coding RNAs analysis, Mir-X™ miRNA First-Strand Synthesis Kit (Takara Clontech Laboratories, Biokom, Poznań, Poland) was used. The procedures were conducted accordingly to manufacturers’ instructions. The cDNA was synthesised from 150 ng of RNA. The qPCR reactions were performed using SensiFAST SYBR®&Fluorescein Kit (Bioline Reagents Ltd., London, UK) and CFX Connected Real-Time PCR Detection System (Bio-Rad, Hercules, CA, USA). The reactions were performed at least in triplicate. The reaction conditions: initial denaturation (95 °C, 2 min) and 45 cycles consisting of denaturation (95 °C, 5 s), annealing (10 s) and elongation (72 °C, 5 s). The melting curve was performed using a gradient protocol (65 to 95 °C, heating rate 0.2 °C/s). RQMAX algorithm was used to calculate the values of transcripts expression. Expression values of *Gapdh* (glyceraldehyde 3-phosphatehydrogenase) and *B2m* (beta-2-microglobulin) genes were used for the purpose of normalisation; however, miRNA expression values were normalised to snU6 gene. The normalisation was performed using the formula: *ΔCt* = *Ct (gene of interest) − Ct (housekeeping gene)*. Among obtained ΔCt values, a maximum value was emerged (MAX value), which was used for standardisation of obtained results. The standardisation and calculation of gene expression was performed using the formula *RQMAX* = *2*^*(MAX value* − *ΔCt)*^. The characterisation of used primers is presented in Table 1. The accession numbers presented in Table 1 refer to specific nucleotides and can be found in the official database of National Center for Biotechnology Information (https://www.ncbi.nlm.nih.gov/).

**Table 1:** The characteristic of primers used in RT-qPCR

Gene	Primer Sequence 5′-3′	Annealing [°C]	Accession No
*Ppar-γ*	**F:**CTCTGCTGGGGATCTGAAGG	58.8	NM_001308354.1
	**R:**GGAATGCGAGTGGTCTTCCA		
*mTOR*	**F:**CTTGGAGAACCAGCCCATAA	60.0	NM_020009.2
	**R:**CTGGTTTCACCAAACCGTCT		
*Lamp2*	**F:**CTTAGCTTCTGGGATGCCCC	60.0	NM_001017959.2
	**R:**TCATCCAGCGAACACTCCTG		
*Mfn-1*	**F:**ATCACTGCAATCTTCGGCCA	60.0	NM_024200.4
	**R:**AGCAGTTGGTTGTGTGACCA		
*Mff*	**F:**TCACATTTGGTGAGTGGGGC	60.0	NM_001372412.1
	**R:**TTTTCCGGGACCCTCATTCG		
*Bcl-2*	**F:**ATCGCCCTGTGGATGACTGAG	58.8	NM_000633.2
	**R:**CAGCCAGGAGAAATCAAACAGAGG		
*Bax*	**F:**ACCAAGAAGCTGAGCGAGTGTC	58.8	NM_001291428.1
	**R:**ACAAAGATGGTCACGGTCTGCC		
*Mmp-9*	**F:**GATGCCAACCTCCTCAACGA	60.0	NM_053056.2
	**R:**GGAAGCGGTCCAGGTAGTTC		
*Runx-2*	**F:**TCCGAAATGCCTCTGCTGTT	58.8	NM_001271630.1
	**R:**GCCACTTGGGGAGGATTTGT		
*Coll-1*	**F:**CAGGGTATTGCTGGACAACGTG	61.4	NM_007742.4
	**R:**GGACCTTGTTTGCCAGGTTCA		
*Opn*	**F:**AGACCATGCAGAGAGCGAG	57.3	NM_001204203.1
	**R:**GCCCTTTCCGTTGTTGTCCT		
*Ocl*	**F:**GGTGCAGACCTAGCAGACACCA	57.0	NM_001032298.3
	**R:**CGCTGGGCTTGGCATCTGTAA		
*Opg*	**F:**AGCCACGCAAAAGTGTGGAA	58.8	NM_008764.3
	**R:**TCCTCTCTACACTCTCGGCA		
*Alpl*	**F:**TTCATAAGCAGGCGGGGGAG	60.0	NM_007431.3
	**R:**TGAGATTCGTCCCTCGCTGG		
*Bmp-2*	**F:**CTACAGGGAGAACACCCGGA	60.0	NM_007553.3
	**R:**GGGGAAGCAGCAACACTAGAA		
*Trap*	**F:**GTCTCTGGGGGACAATTTCTACT	60.0	XM_006509945.3
	**R:**GTTTGTACGTGGAATTTTGAAGC		
*Ctsk*	**F:**TAACAGCAAGGTGGATGAAATCT	60.0	NM_011613.3
	**R:**CTGTAGGATCGAGAGGGAGGTAT		
*Nfatc-1*	**F:**TTCGAGTTCGATCAGAGCGG	60.0	NM_001164112.1
	**R:**AGGTGACACTAGGGGACACA		
*Pu.1*	**F:**GAGAAGCTGATGGCTTGGAG	60.0	NM_001378899.1
	**R:**TTGTGCTTGGACGAGAACTG		
*Gapdh*	**F:**GTCAGTGGTGGACCTGACCT	58.8	NM_001289746.1
	**R:**CACCACCCTGTTGCTGTAGC		
*B2m*	**F:**CATACGCCTGCAGAGTTAAGCA	58.8	NM_009735.3
	**R:**GATCACATGTCTCGATCCCAGTAG		
*miR-7a-5p*	TGGAAGACTAGTGATTTTGTTGT	58.8	MIMAT0000677
*miR-17-5p*	CAAAGTGCTTACAGTGCAGGTAG	58.8	MIMAT0000649
*miR-21a-5p*	TAGCTTATCAGACTGATGTTGA	58.8	MIMAT0000530
*miR-124-3p*	TAAGGCACGCGGTGAATGCC	58.8	MIMAT0000134
*miR-145-5p*	GTCCAGTTTTCCCAGGAATCCCT	58.8	MIMAT0000437
*miR-203a-3p*	GTGAAATGTTTAGGACCACTAG	58.8	MI0000283
*miR-223-3p*	TGTCAGTTTGTCAAATACCCCA	58.8	MIMAT0000280

### The effectiveness of new bone formation in vivo

The in vivo study was conducted with the full approval of the Local Ethics Committee for Animal Experiments in Wroclaw (Resolution no.069/2020/P1, 9.12.2020). The guidelines included in the Act on the Protection of Animals Used for Scientific or Educational Purposes from 15 of January 2015, which implements Directive 2010/63/EU of the European Parliament and the Council of 22 September 2010, were fully followed during the study. Moreover, the procedures of PN-EN ISO 10993–2:2006 standards were used. Senescence accelerated osteoporotic SAM/P6 mice were purchased from Envigo (Indianapolis, IN, USA). One-week period of acclimatisation was preceded before the experiment. The mice were housed under a 12 h light/dark cycle with constant temperature (22 ± 2 °C) and humidity (50 ± 10%), as well as fed with a standard chow diet and ad libitum access to water.

A bilateral cranial defect was performed to assess the osteoinductive properties of miR-21. The procedure of cranial defect model conduction was described previously and performed in two (n = 2) SAM/P6 mice [30]. The procedures were performed under the supervision of a veterinarian. The animals were subjected to general anaesthesia with the mixture of xylazine (25 mg/kg) and ketamine (70 mg/kg) and placed in a prone position. The type of anaesthesia was recommended due to the severe nature of the procedure and to minimise the suffering of the animals. The operating site was disinfected with a chlorhexidine solution. Then, the scalp and the periosteum on both sides of the parietal bone were incised. The periosteum was removed to expose the skull bone. The circular cranial defect (2 mm in diameter) was drilled using a cylindrical low-speed carbide bur with 1 mm of diameter. In order to deliver undegraded miR-21 to the defect site, a fully biocompatible and biodegradable biomaterial based on sodium alginate was prepared. Briefly, 2% solution of sodium alginate was prepared in NaCl and filtrated using 0.45 and 0.22 µm syringe filters. Simultaneously, 0,5 M solution of CaCl_2_ was prepared. MISSION® miRNA miR-21 Mimic (HMI0371, Sigma-Aldrich, Munich, Germany) was added to the sodium alginate solution at the concentration 50 nM and mixed for 10 s using the vortex. Shortly before the procedure, the sodium alginate and CaCl_2_ solution were mixed in a ratio 1:1 in order to synthesise a hydrogel. The alginate-based biomaterials with 50 nM of miR-21 were allocated in the right defect sites during the procedure. Left defect sites serve as controls, where hydrogels without miR-21 were administered. The procedure was performed in the presence of a heat lamp and the cuts were closed with 6–0 synthetic seams. Every 12 h, for three days after surgery, animals received painkillers i.e. buprenorphine (0.03 mg/kg) and meloxicam (1 mg/kg). Further, mice were sacrificed two weeks after the cranial defect procedure to analyse the formation of novel bone tissue. Before the sacrifice procedure, the animals were given an aesthetic (mixture of xylazine—15 mg/kg and ketamine—50 mg/kg) in order to minimalize the stress and suffering of animals. 15 min after administration of anaesthetics, animals were euthanized by the disruption of the spinal cord. The procedure was performed by a person qualified to kill animals. The death of each animal was confirmed by a veterinarian.

Mice skulls were dissected for the µ-CT and SEM–EDX analyses. The computed microtomography measurements were performed in the X-ray Microtomography Laboratory at the Faculty of Computer Science and Materials Science, University of Silesia in Katowice (Chorzów, Poland) using the GE Phoenix v|tome|x microtomography system (General Electric, Cincinnati, OH, USA) at voltage 140 kV and current intensity 40 μA. The voxel size was 5.5 μm3. Detector sensitivity × 2 and binning × 1. Tomographic reconstructions were based on projection of 1800 images equally spaced through 360º (exposure time 500 ms). The µ-CT scans were exported as VGL files and analysed using myVGL software (version 3.3.2.170119, Volume Graphic GmbH) in order to assess the structural properties of newly formed tissue. The initial resolution of obtained images was: 652 × 456 pixels. For evaluating new tissue contribution, Fiji is just ImageJ Software was used. Moreover, the skulls were analysed using scanning electron microscopy with energy-dispersive X-ray analysis (SEM–EDX; SEM Evo LS 15 Zeiss, Germany) operating at 10 000 V and work distance 11 mm. The photographs of the defect sites were taken under 35 × magnification. The images resolution was: 1024 × 768 pixels. The mapping of calcium, phosphorus and hydroxyapatite composition was performed using Bruker Quantax 200 System with BrukerXFlash 5010 detector and Esprit 1.8 Software. Each analysis was performed under 35 × magnification for 120 s operating at 20 000 V and a working distance set at 8.5 mm. Furthermore, the expression of crucial markers related to proper bone regeneration was examined using RT-qPCR technique as described in the previous paragraph.

### Statistical analyses

Each analysis was performed in three technical repetitions. The material for the ex vivo experiments was delivered from 15 BALB/c and 15 SAM/P6 mice. The statistical analyses were performed using GraphPad Prism 5 (GraphPad Software, San Diego, CA, USA). The data were obtained using one-way analysis of variance (ANOVA) with Tukey’s post hoc test. Differences were considered as statistically significant at *p* < 0.05. The significance levels were indicated with asterisks: *p < 0.05, **p < 0.01, ***p < 0.001. Non-significant differences were marked as *ns*.

## Results

### MiR-21 improves viability and proliferative activity in senile osteoporotic BMSCs

**Fig. 1:**
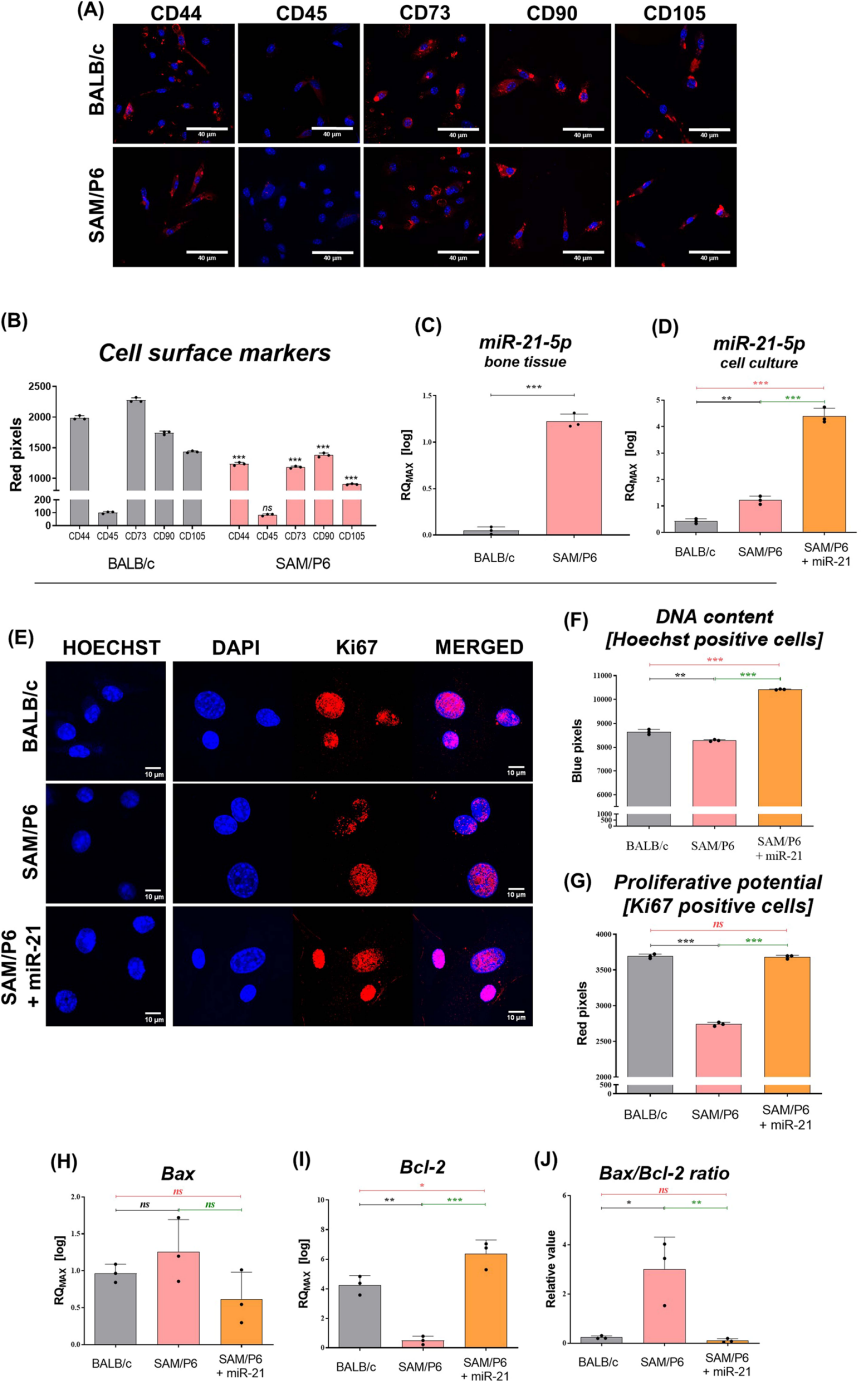
MiR-21 improves viability and proliferative activity in BMSCs isolated from senile osteoporotic SAM/P6 mice. **A** The representative photographs (z-stack) of cells surface markers (CD44, CD45, CD73, CD90 and CD105) in BMSCs isolated from BALB/c and SAM/P6 mice. The staining intensity of visualised antigens was presented as bar graph (**B**). The level of miR-21-5p was analysed in bone tissue samples delivered from BALB/c and SAM/P6 mice (**C**), as well as in cell cultures after transfection with miR-21 mimic (**D**). **E** The representative photographs (z-stacks) of cells stained with Hoechst 33342 and Ki67 protein in BMSCs isolated from BALB/c and SAM/P6 mice. The staining intensity of visualised **F** nuclei and **G** Ki67 protein were presented as bar graphs. The RT-qPCR technique was used to present the gene expression of **H** *Bax*, **I** *Bcl-2* and **J** *Bax/Bcl-2* ratio. The RT-qPCR measurements were performed using RQMAX method and presented in a log scale. The confocal microscope photographs were taken under 630-fold & 1000-fold magnification and the scale bars are equal to 40 µm and 10 µm, respectively. Significant differences between groups are indicated with asterisk: *p < 0.05, **p < 0.01, *** and *p* < 0.001. Non-significant differences are marked as *ns*

We confirmed the presence of BMSC population based on the expression of cell surface antigens typical for mesenchymal stem cells. Obtained cultures were characterised by accumulation of CD44, CD73, CD90 and CD105, while there was no presence of CD45 marker (Fig. 1A). Importantly, the accumulation of presented antigens was reduced in BMSCs delivered from osteoporotic SAM/P6 mice (Fig. 1B). The loss of the expression of cell surface markers may be correlated with senescent-like phenotype of BMSC_SAM/P6_. Further, the miR-21-5p level was assessed in bone tissue collected from BALB/c and SAM/P6 mice, as well as in BMSCs. We noticed a higher transcript level of miR-21 in SAM/P6 mice when compared to BALB/c in both bone tissue and cells samples (Fig. 1C, D). It is known that non-coding RNAs are one of many factors that affect the cells status, thus does not direct their biology independently. Although miR-21-5p expression was noticed to be upregulated in BMSCs and bones delivered from SAM/P6 mice, the activity of other crucial markers (senile phenotype, high activity of osteoclasts) affects the bone homeostasis that finally leads the mice into osteoporotic state. In addition, we confirmed the transfection efficiency that was observed by upregulation of miR-21-5p expression in the treated BMSC_SAM/P6+miR-21_ comparing to non-transfected BMSC_SAM/P6_ (Fig. 1D). Senile osteoporotic BMSC_SAM/P6_ were characterised by decreased DNA level (Hoechst positive cells) that was additionally manifested by deteriorated proliferative potential (Ki67 positive cells) when compared to healthy BMSC_BALB/c._ However, it was noted that miR-21 increased the DNA level, thus improving proliferative potential of BMSC_SAM/P6+miR-21_ (Fig. 1E–G). Furthermore, miR-21 improved the viability of BMSC_SAM/P6+miR-21_ evidenced by downregulation of *Bax/Bcl-2* ratio (Bcl-2 associated X protein / B-cell lymphoma 2) (Fig. 1H–J). *Bax* and *Bcl-2* are both key players during intrinsic apoptotic pathway triggered by mitochondrial dysfunction. It was shown that *Bax* initialise cell death, while *Bcl-2* prevents apoptosis by inhibiting the activity of *Bax*.

### MiR-21 reduces autophagy and regulates mitochondria dynamics in senile osteoporotic BMSCs

**Fig. 2:**
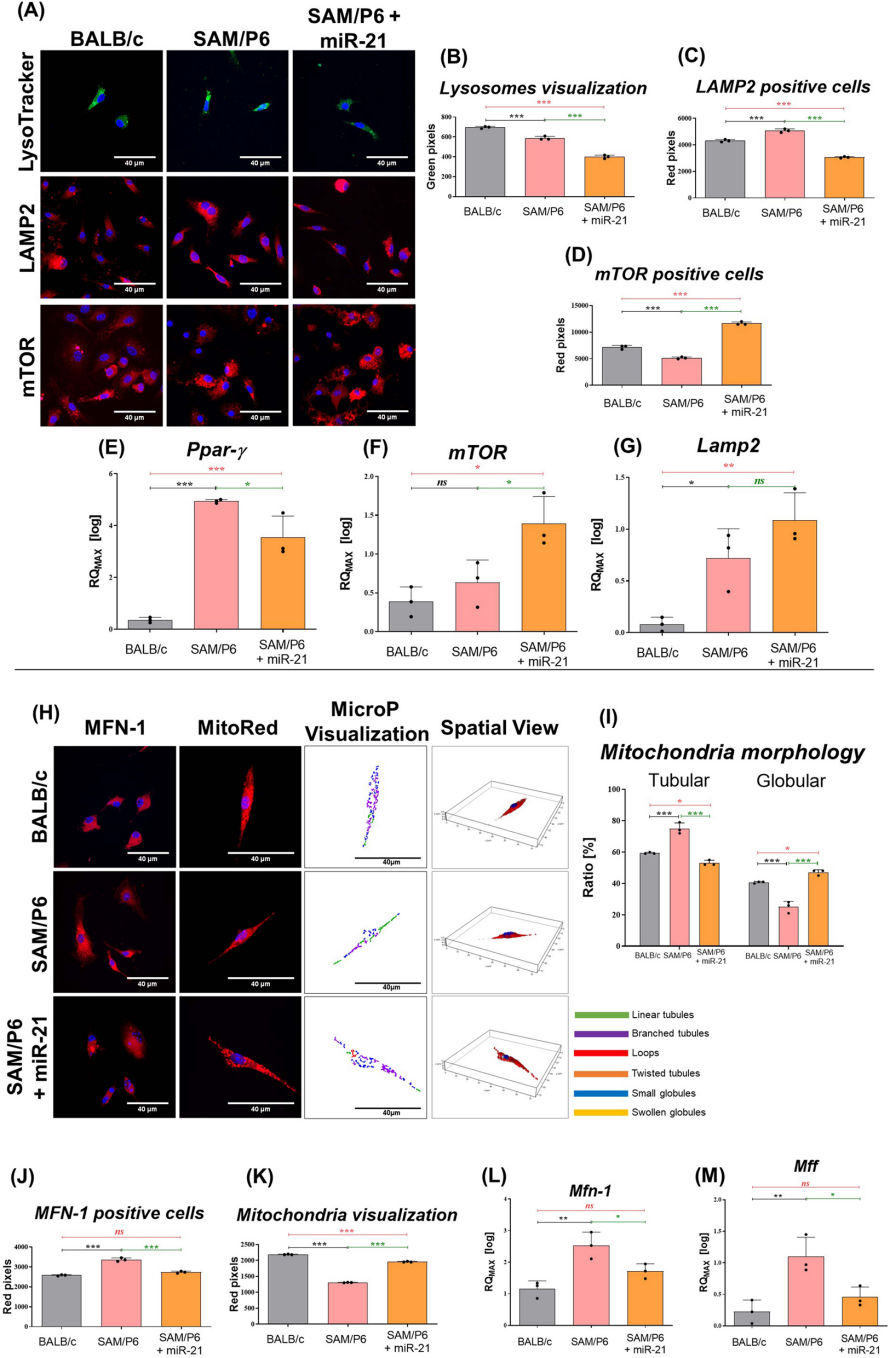
MiR-21 reduces autophagy and regulates mitochondria dynamics in BMSCs isolated from senile osteoporotic SAM/P6 mice. **A** The representative photographs (z-stacks) of cells’ nuclei stained with lysosomes, LAMP2 and mTOR protein in BMSCs isolated from BALB/c and SAM/P6 mice. The staining intensity of visualised **B** lysosomes, **C** LAMP2 protein and **D** mTOR protein were presented as bar graphs. The RT-qPCR technique was used to present the gene expression of **E** *Ppar-γ*, **F** *mTOR* and **G** *Lamp2*. **H** The representative photographs (z-stacks) of nuclei with stained MFN-1 protein and mitochondrial network in BMSCs isolated from BALB/c and SAM/P6 mice. The images were supplemented with MicroP visualisation of mitochondria morphology and presented as a bar graph (**I**). The staining intensity of visualised **J** MFN-1 protein and **K** mitochondria network were presented as bar graphs. The RT-qPCR technique was used to present the gene expression of **L** *Mfn-1* and **M** *Mff*. The RT-qPCR measurements were performed using RQMAX method and presented in a log scale. The confocal microscope photographs were taken under 630-fold and 1000-fold magnification and the scale bar is equal to 40 µm. Significant differences between groups are indicated with asterisk: *p < 0.05, **p < 0.01, ***p < 0.001. Non-significant differences are marked as *ns*

Obtained results demonstrated that upregulated expression of miR-21 in senile osteoporotic BMSC_SAM/P6_ impedes autophagy in bone marrow-derived mesenchymal stem/stromal cells. It has been evidenced by the decreased intensity of lysosomes and LAMP2 (lysosome-associated membrane protein 2) protein staining but greater intensity of mTOR (mammalian target of rapamycin kinase) staining in BMSC_SAM/P6+miR-21_, when compared to BMSC_SAM/P6_ (Fig. 2A–D). Further, it has been noticed that BMSC_SAM/P6_ were characterised by upregulated expression of *PPAR-γ* (peroxisome proliferator-activated receptor gamma) and *Lamp2* (Fig. 2E, G), which serve as markers of selective autophagy. Notably, the expression of *PPAR-γ* was significantly downregulated in BMSC_SAM/P6+miR-21_ compared to BMSC_SAM/P6_ (Fig. 2E). Moreover, the expression level of *mTOR* in osteoporotic BMSC was elevated after miR-21 upregulation (Fig. 2F). It is known that the activation of mTOR pathway inhibits the pathological induction of autophagy. No significant changes were observed in *Lamp2* expression (Fig. 2G).

The presence of miR-21 within BMSCs cultures significantly regulated the dynamic of the mitochondrial network, which was evidenced by the modulation of fusion and fission processes. In senile osteoporotic BMSC_SAM/P6_, the mitochondria were characterised by their tubular-shape morphology observed in the senescent cells. However, after miR-21 upregulation, the mitochondria underwent fission processes evidenced by the decreased percentage of tubular-shape mitochondria in favour of globular-shape mitochondria (Fig. 2H, I). This crucial finding indicates the ongoing selective macroautophagy (mitophagy), which is responsible for degradation of defective mitochondria and maintain cellular homeostasis. Moreover, mitochondrial network activity reflected by a signal from MitoRed staining revealed that osteoporotic BMSC_SAM/P6_ show signs of depolarization. However, miR-21 upregulation improved mitochondrial activity, which was correlated with an enhanced MitoRed signal. Simultaneously reduced signal intensity of stained MFN-1 protein (mitofusin-1), as well as downregulated *Mfn-1* and *Mff* (mitochondria fission factor) expression, were characteristic for BMSC_SAM/P6+miR-21_, compared to BMSC_SAM/P6_ (Fig. 2H, J, K, L, M).

The complementary results regarding the modifications of mitochondria phenotype as a result of miR-21 upregulation were presented in Additional file 1: Figure S1. The prevalence of elongated and tubular-shaped mitochondria corresponded to their low number noted in BMSC_SAM/P6_. The upregulation of miR-21 increased the number of mitochondria that stayed in line with the modification of mitochondria phenotype (Additional file 1: Figure S1A). The detailed analysis of fission and fusion processes has shown that BMSC_SAM/P6_ were characterised by an increased occurrence of mitochondria classified as simple tubes simultaneously accompanied by a lowered ratio of branching tubes and small globules when compared to BMSC_BALB/c_. The addition of miR-21 drove the mitochondria reconstruction, evidenced by a decreased percentage of simple tubes, while upregulated branching tubes and small globules (Additional file 1: Figure S1B–D).

### MiR-21 improves osteogenic differentiation in senile osteoporotic BMSCs

**Fig. 3:**
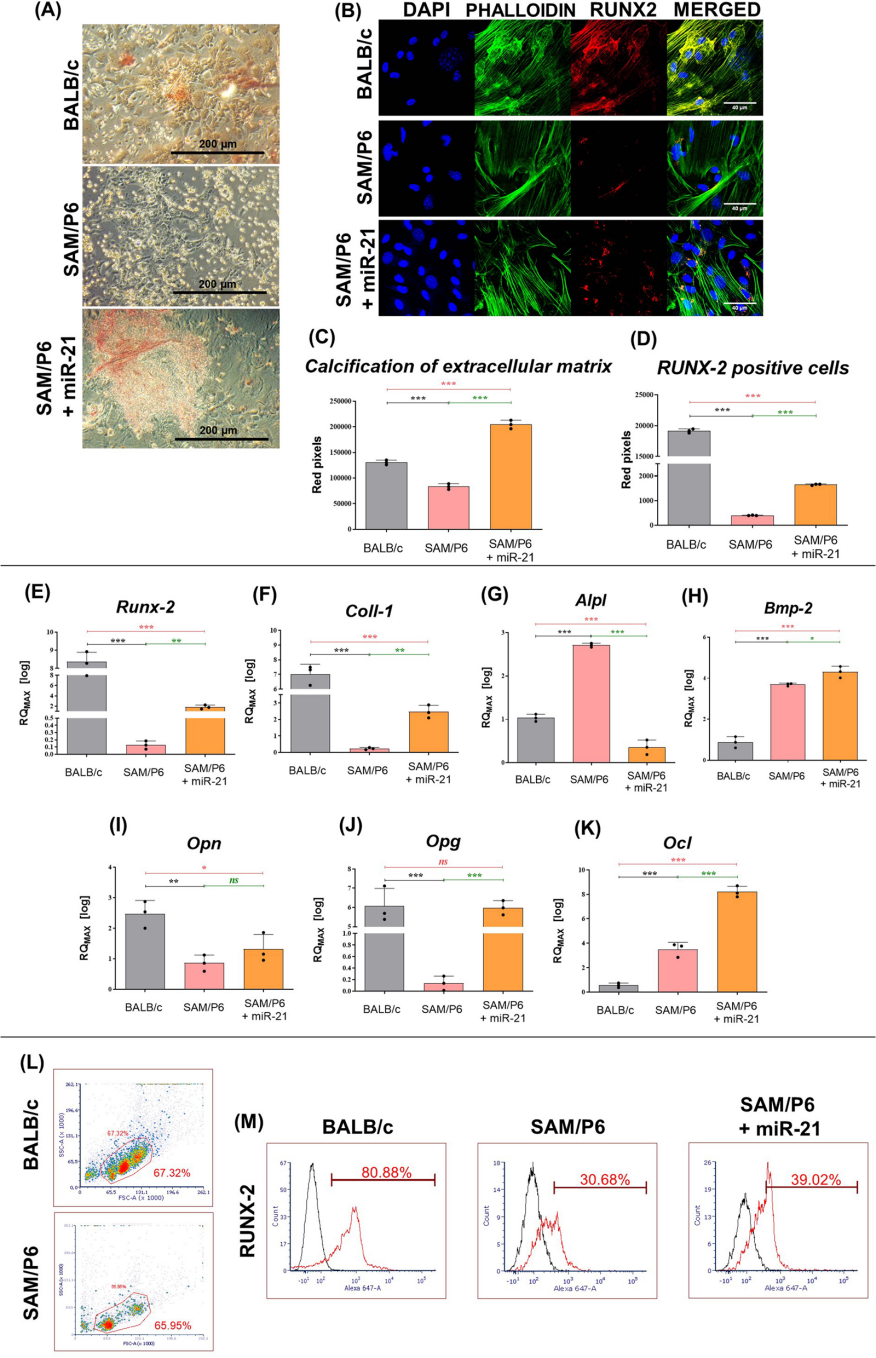
MiR-21 improves osteogenic differentiation of BMSCs isolated from senile osteoporotic SAM/P6 mice. **A** The representative images of BMSCs isolated from BALB/c and SAM/P6 mice cultured under osteogenic conditions. The images were taken using an inverted light microscope under 100-fold magnification and the scale bar is equal 200 µm. The staining signals of visualised calcium deposits were presented as a bar graph (**C**). **B** The representative images (z-stacks) of cells’ nuclei, actin cytoskeleton and RUNX-2 protein in BMSCs isolated from BALB/c and SAM/P6 mice. The confocal microscope photographs were taken under 630-fold magnification and the scale bar is equal to 40 µm. The staining intensity of RUNX-2 protein was presented as a bar graph (**D**). The RT-qPCR technique was used to present the gene expression of **E** *Runx-2*, **F** *Coll-1*, **G** *Alpl*, **H** *Bmp-2*, **I** *Opn*, **J** *Opg* and **K** *Ocl*. The RT-qPCR measurements were performed using RQMAX method and presented in a log scale. **L** The gating procedure of BMSCs isolated from BALB/c and SAM/P6 mice. **M** The histograms presenting the positively stained population of cells (red line) for RUNX-2 protein. Each measurement was performed for 1000 events. Significant differences between groups are indicated with asterisk: *p < 0.05, **p < 0.01, ***p < 0.001. Non-significant differences are marked as *ns*

The extracellular matrix (ECM) composition after BMSCs osteogenic differentiation indicated lowered osteogenic potential of senile BMSC_SAM/P6_ compared to healthy BMSC_BALB/c_. However, improved quantity and quality of calcium deposits in BMSC_SAM/P6+miR-21_ cultures showed pro-osteogenic properties of miR-21 (Fig. 3A, C). At the same time, the actin cytoskeleton was poorly developed in BMSC_SAM/P6_ cultures which led to impaired cell–cell contact, lowered cells’ confluency and finally deteriorated differentiation. Moreover, the signal intensity of stained RUNX-2 (runt-related transcription factor 2) protein was poorer in BMSC_SAM/P6_, when compared to BMSC_BALB/c._ The upregulation of miR-21 improved the development of the actin cytoskeleton in osteoporotic BMSCs and caused an increased protein expression of RUNX-2 during osteogenic differentiation (Fig. 3B, D). Importantly, miR-21 affected the expression of crucial osteogenic markers during BMSCs differentiation. Osteoporotic BMSC_SAM/P6_ were characterised by lower expression of *Runx-2* and *Coll-1* (collagen type 1), but elevated transcripts level of *Alpl* (alkaline phosphatase) and *Bmp-2* (bone morphogenetic protein 2), which serve as early osteogenic markers. The upregulation of miR-21 increased the expression of *Runx-2*, *Coll-1* and *Bmp-2* in BMSC_SAM/P6+miR-21_, but downregulated the transcript level of *Alpl* (Fig. 3E, F, G, H). Additionally, BMSC_SAM/P6_ expressed less transcripts of late osteogenic markers, such as *Opn* (osteopontin) and *Opg* (osteoprotegerin), when compared to BMSC_BALB/c_. Interestingly, BMSC_SAM/P6_ were also characterised by higher expression of *Ocl* (osteocalcin). Upregulation of miR-21 promoted osteogenesis in BMSC_SAM/P6_ which was manifested by increased expression of *Opn*, *Opg* and *Ocl* transcripts (Fig. 3I–K). Moreover, cytometric evaluation of isolated bone marrow cells revealed that miR-21 upregulates the expression of RUNX-2 protein in BMSC_SAM/P6_ (Fig. 3L, M).

### MiR-21 inhibits osteoclastogenesis in senile osteoporotic BMSCs

**Fig. 4:**
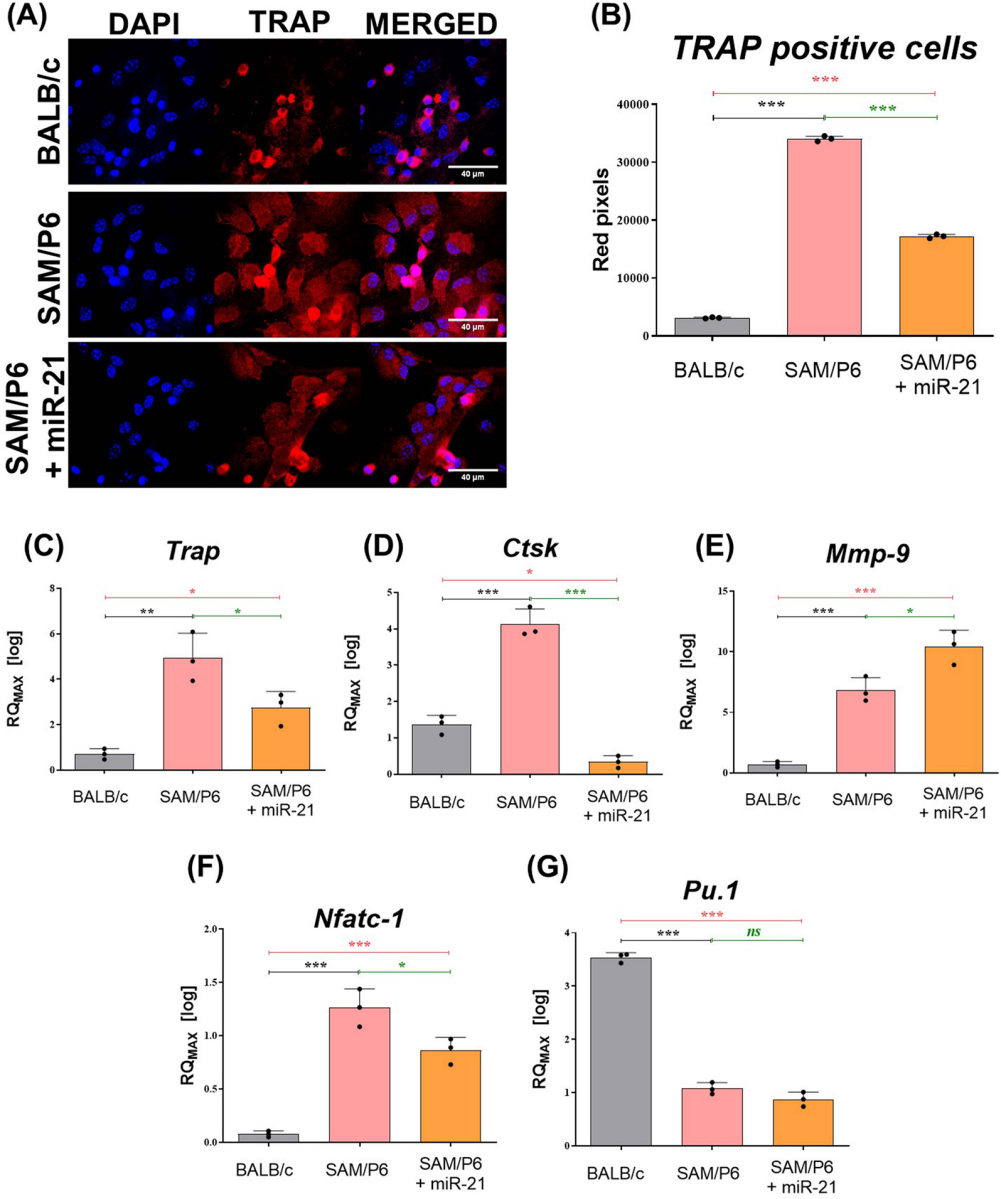
MiR-21 inhibits osteoclastogenesis in BMSCs isolated from senile osteoporotic SAM/P6 mice. **A** The representative images (z-stacks) of cells’ nuclei, actin cytoskeleton and TRAP protein in BMSCs isolated from BALB/c and SAM/P6 mice. The confocal microscope photographs were taken under 630-fold magnification and the scale bar is equal to 40 µm. The staining intensity of TRAP protein was presented as a bar graph (**B**). The RT-qPCR technique was used to present the gene expression of **C** *Trap*, **D** *Ctsk*, **E** *Mmp-9*, **F** *Nfatc-1* and **G** *Pu.1*. The RT-qPCR measurements were performed using RQMAX method and presented in a log scale. Significant differences between groups are indicated with asterisk: *p < 0.05, **p < 0.01, ***p < 0.001. Non-significant differences are marked as *ns*

It has been shown that miR-21 inhibits the maturation of osteoclasts and impede the expression of osteoclastic markers. After miR-21 upregulation, the signal of TRAP-positive cells during microscopic analyses in osteoporotic BMSCs was significantly reduced (Fig. 4A, B). Moreover, after miR-21 upregulation, the expression of *Trap*, *Ctsk* (cathepsin K) and *Nfatc-1* (nuclear factor of activated T-cells, cytoplasmic 1) in osteoporotic BMSCs was significantly decreased (Fig. 4C, D, F). Simultaneously, the level of *Pu.1* (transcription factor PU.1) in BMSC_SAM/P6+miR-21_ was downregulated (Fig. 4G), but *Mmp-9* (matrix metalloproteinase 9) level was upregulated (Fig. 4E), when compared to BMSC_SAM/P6_.

### MiR-21 affects the expression of non-protein-coding RNAs in BMSCs isolated from senile osteoporotic SAM/P6 mice

**Fig. 5:**
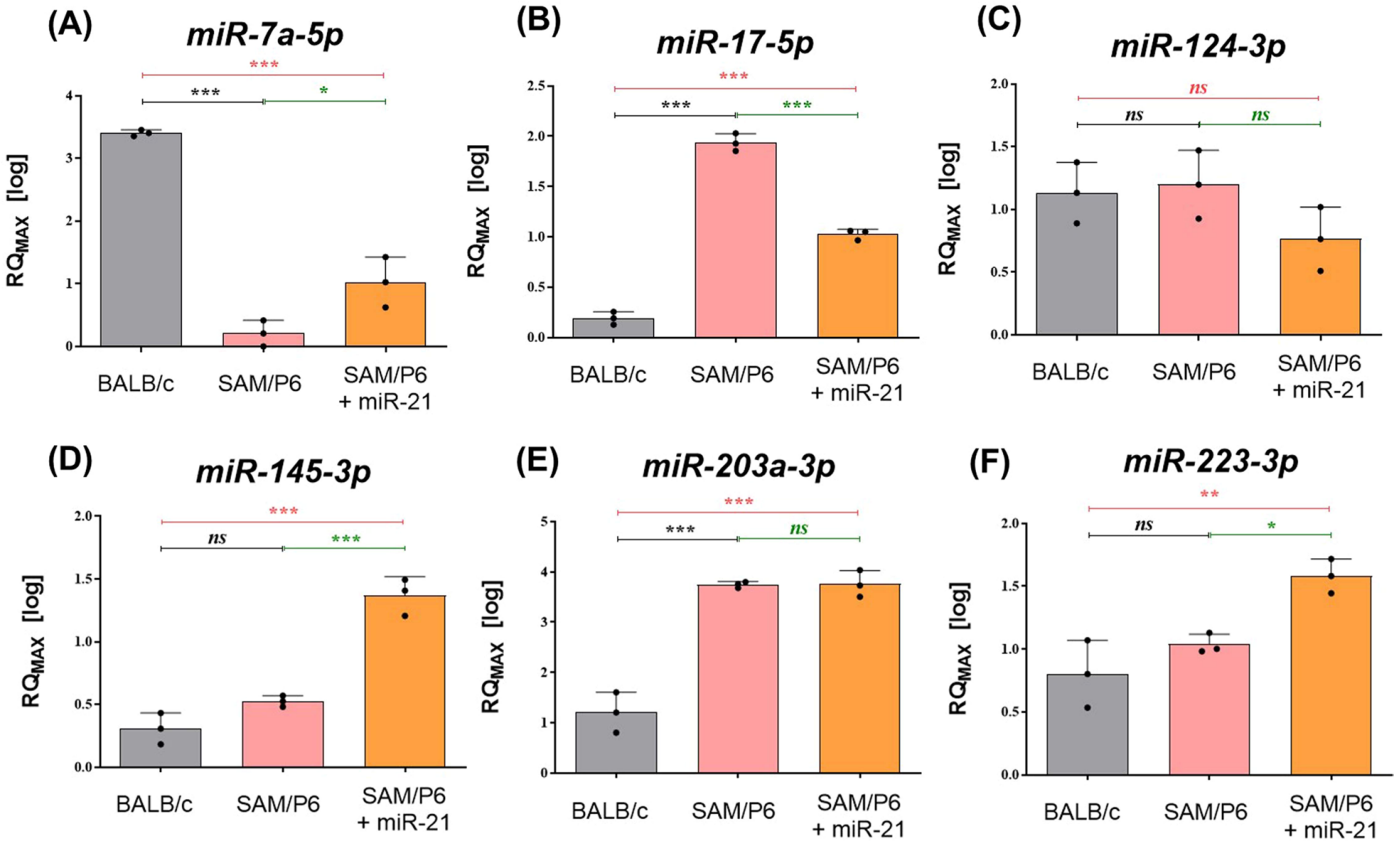
MiR-21 affects the expression of non-protein-coding RNAs in BMSCs isolated from senile osteoporotic SAM/P6 mice. The RT-qPCR technique was used to present the gene expression of **A** *miR-7a-5p*, **B** *miR-17-5p,* **C** *miR-124-3p*, **D** *miR-145-3p*, **E** *miR-203a-3p* and **F** *miR-223-3p*. The RT-qPCR measurements were performed using RQMAX method and presented in a log scale. Significant differences between groups are indicated with asterisk: *p < 0.05, **p < 0.01, ***p < 0.001. Non-significant differences are marked as *ns*

It has been noticed that miR-21 promotes the osteogenic potential of senile osteoporotic BMSCs, thus we decided to evaluate if miR-21 affects the miRNAs essential for bone homeostasis. After miR-21 upregulation, the level of *miR-7a-5p*, *miR-145-3p* and *miR-223-3p* was significantly elevated (Fig. 5A, D, F). At the same time, the level of *miR-17-5p* went down (Fig. 5B). The level of *miR-124-3p* and *miR-203a* did not change significantly (Fig. 5C, E).

### MiR-21 improves bone regeneration in senile osteoporotic SAM/P6 mice

**Fig. 6:**
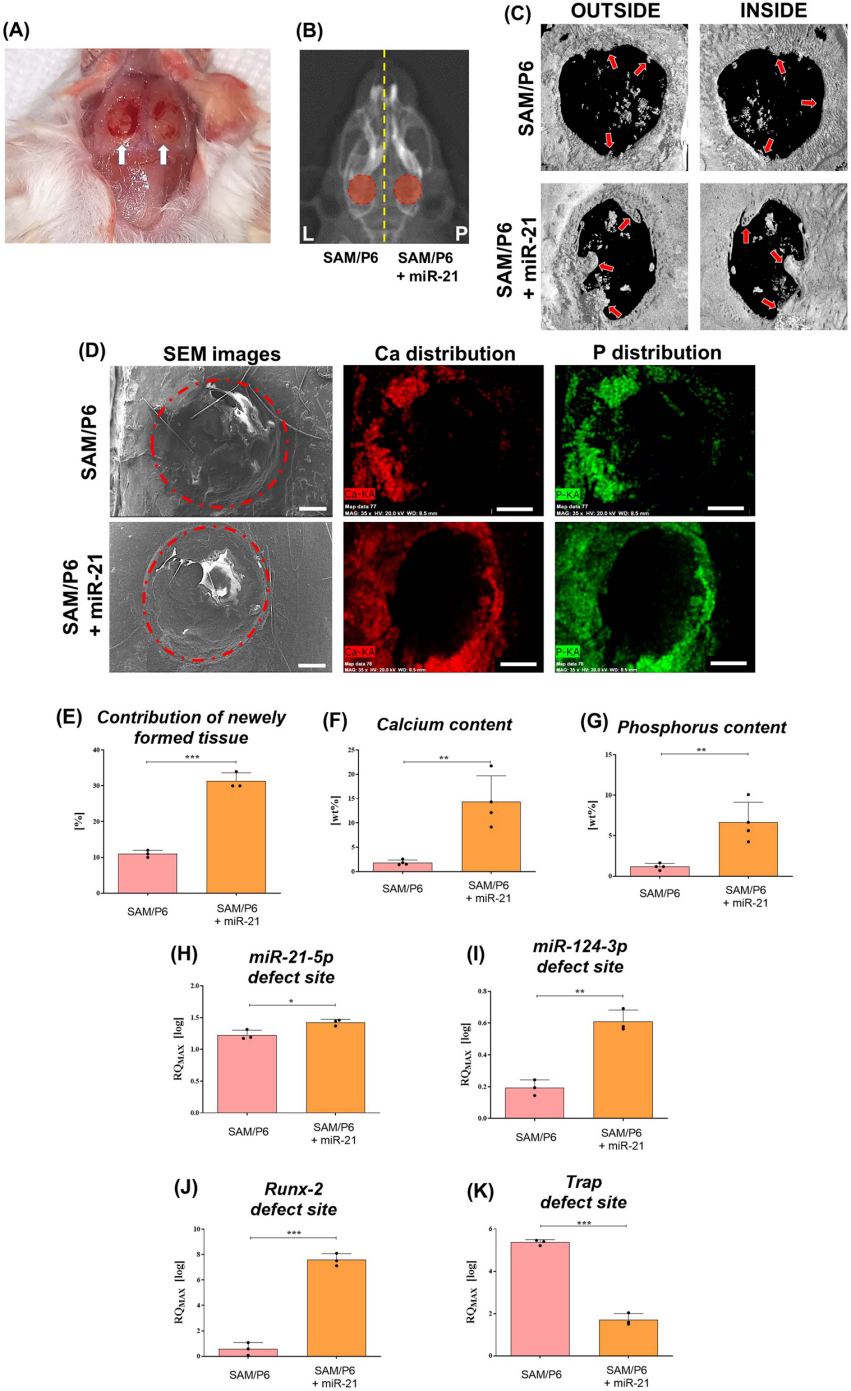
MiR-21 improves regenerative potential of BMSCs in senile osteoporotic SAM/P6 mice strain in vivo. During the procedure, two mice (n = 2) were operated. **A** The photograph of critical-size cranial defects during the mice operation. **B** The RTG images of SAM/P6 mice 2 weeks after the procedure. The defects were marked on the images using red circles. Scale bar is equal to 5 mm. **C** The external and internal photographs obtained during µCT analyses of the defects in SAM/P6 mice. The ratio of newly formed tissue to the initial defect area was presented as a bar graph (**E**) and the new bone was indicated with red arrows. **D** The SEM–EDX analyses of the defects, as well as calcium and phosphorus distribution in SAM/P6 mice. The images were captured under 35-fold magnification and the scale bar is equal to 200 µm. The SEM–EDX signal of calcium and phosphorus concentration were measured and presented as bar graphs (**F**, **G**). The mRNA level of **H** *miR-21-5p*, **I** *miR-124-3p*, **J** *Runx-2* and **K** *Trap* within the defect site. Significant differences between groups are indicated with asterisk: *p < 0.05, **p < 0.01, ***p < 0.001. Non-significant differences are marked as *ns*

The in vivo study performed using senile osteoporotic SAM/P6 mice strain confirmed the pro-osteogenic properties of miR-21. The fully biocompatible complexes consisted of 5% sodium alginate functionalized with miR-21 were placed in the centre of parietal bones (Fig. 6A, B). Two weeks after the operation, SEM–EDX analyses revealed elevated concentrations of phosphorus (P) and calcium (Ca) within defect sites after miR-21 application (Fig. 6D, F, G). Importantly, X-ray computed microtomography (µ-CT) analyses indicated the higher contribution of newly created bone tissue in SAM/P6 mice after miR-21 treatment. (Fig. 6C, E). In addition, the transcript levels of *miR-21-5p*, *miR-124-3p* and *Runx-2* were upregulated, while *Trap* was downregulated in the defect site. Obtained results were comparable to analyses using BMSCs model and indicated enhanced bone regeneration after miR-21 application (Fig. 6H–K).

## Discussion

Osteoporosis is reaching epidemic levels among the elderly worldwide, leading to serious health complications, including bone fractures [31]. Numerous molecular pathways have been identified to play a critical role in regulating osteoporosis, including those regulated by miRNAs [32, 33]. In this study, for the first time, we have investigated the rejuvenating role of miR-21-5p in the course of senile osteoporosis. We have determined the influence of miR-21 upregulation on mitochondria network dynamics as well as impaired osteogenic differentiation of BMSCs isolated from osteoporotic SAM/P6 senile mice. Previously, miR-21-5p was widely considered a potential diagnostic and therapeutic tool that may find application during skeletal system disabilities, including osteoporosis-related fractures [20, 28, 34, 35]. Here, we have discovered that miR-21-5p drives the mitochondria dynamic, reversing the senile phenotype and improving the osteogenic differentiation potential of osteoporotic mice derived BMSC_SAM/P6_, as well as promote bone regeneration in tissue milieu in a bilateral cranial defect in SAMP/P6 mice.

Our previous study showed that BMSC_SAM/P6_ are characterised by impaired multilineage differentiation potential, increased senescence, lowered stemness, as well as decreased proliferative activity, confirming the diseased phenotype of BMSCs isolated from osteoporotic patients [25, 36]. Additionally, the senescent BMSCs are characterised by depolarization of mitochondrial membrane that affects disturbed metabolic activity and clearly indicates an impaired ability of SAM/P6 derived BMSCs to produce a properly mineralized extracellular matrix. For that reason, there is a huge need to investigate the molecular mechanisms that must be improved in order to restore BMSCs proper functions. Here, we have shown that miR-21-5p upregulated viability and proliferative potential of senile osteoporotic BMSCs. It was evidenced by decreased *Bax/Bcl-2* ratio, as well as a greater accumulation of Ki67 protein, which serves as a marker of actively dividing cells [37]. Moreover, the confocal analysis of the accumulation of cell surface markers, such as CD44, CD73, CD90 and CD105, confirmed the senile-like phenotype and loss of “stemness” in BMSCs delivered from SAM/P6 mice [38–42].

Further, we confirmed that miR-21-5p significantly regulates the dynamics of the mitochondria network, evidenced by the intensification of their fission processes. The ratio of elongated tubular-shape mitochondria decreased in favour of globular-shape mitochondria in BMSC_SAM/P6+miR-21_, compared to osteoporotic BMSC_SAM/P6_. More specifically, the percentage of distinguished mitochondria subtypes i.e. simple tubes, branching tubes and small globules in BMSC_SAM/P6+miR-21_ became identical to healthy BMSC_BALB/c_. The reconstruction of mitochondria morphology was also noted in the general mitochondria quantity in examined BMSCs. Interestingly, the expression of both *Mfn-1* (mitofusin-1) and *Mff* (mitochondrial fission factor), the master regulators of mitochondria’s fission and fusion, became downregulated. Mfn-1 and Mfn-2 mediate the fusion of outer mitochondria membrane, while the optic atrophy protein 1 (OPA1) mediates the fusion of inner membrane. Although the mitochondria fission is mediated by DRP1 (dynamin-related GTPase), it is recruited through four transmembrane receptors: MFF, FIS1 (mitochondrial fission 1 protein), MiD49 and MiD50 (mitochondrial kinetic protein 49 and 50). Essentially, the mitochondria division contributes to ensure their proper quality that allows to maintain the cells in a healthy state [43]. It has been proven that the presence of fragmented (globular) mitochondria is characteristic for vital and pluripotent cells with high self-renewal potential [44]. Moreover, miR-21-5p impedes autophagy processes in BMSC_SAM/P6_, evidenced by decreased accumulation of lysosomes and LAMP2 protein. The high activity of lysosomes, accompanied with LAMP2 accumulation, is widely associated with the progression of selected autophagy [45, 46]. In addition, the level of *Ppar-γ* was downregulated, while expression of *mTOR* raised, which confirms the potential of miR-21 in autophagy regulation [45]. Previously, it was proven that the activation of mTOR pathway inhibits autophagy induction and prevents pathological expression of lysosomal and autophagy genes [47, 48]. Moreover, the PI3K/Akt/mTOR pathway plays a crucial role in regulating the cell cycle. Its overregulation by miR-21 may release the BMSCs from cell cycle arrest while promotes the cells proliferation.

We have found significantly enhanced expression of *Runx-2, Coll-1, Bmp-2, Opg* and *Ocl* in differentiated BMSC_SAM/P6_ with upregulated miR-21. It seems that miR-21-5p activates both early and late osteogenic-related markers. RUNX-2 has been previously shown to be a master regulator of osteoblast differentiation, matrix production and mineralization, making it a critical early osteogenesis marker [49]. Moreover, RUNX-2 regulates other major osteoblasts specific downstream transcripts, including COLL-1, OPN and OCL. Obtained data strongly correlates with enhanced extracellular matrix formation accompanied with calcium deposits accumulation in BMSC_SAM/P6+miR-21_. The presented results stay in line with our previous studies regarding the pro-osteogenic activity of miR-21. We have shown that downregulation of miR-21 hampers the proper extracellular matrix reconstruction during osteogenic differentiation in murine MC3T3-E1 cell line [20]. Conversely, upregulation of miR-21 combined with miR-124 and nanohydroxyapatite (nHAp), improves the osteogenic differentiation of MC3T3-E1 cells and murine BMSCs [28, 50]. Positive effect of miR-21-5p on osteogenesis in BMSC process has also been confirmed by other authors that found activation of Smad7-Smad1/5/8-Runx-2 pathway in miR-21-KO mice model [51]. Interestingly, recent data indicate a beneficial role of miR-21 as a regulator of osteogenic differentiation of periodontal ligament stem cells by targeting Smad5 [52]. On the other hand, OPG belongs to the tumour necrosis factor receptor (TNFR) superfamily and its inhibitory effect on osteoclasts proliferation and maturation has been shown previously [53]. Moreover, recent data suggest that OPG knockout mice develop osteoporosis and are characterised by an enhanced number of osteoclasts. Furthermore, their BMSCs exhibit high expression of adipogenic related markers leading to accumulation of adipocytes within bone marrow [53]. Contrary, the *Alpl* (alkaline phosphatase) level was downregulated after miR-21 addition. Alpl is considered as a crucial marker of early osteogenesis. Therefore, we hypothesise that it might be a signal of accelerated differentiating processes proven by a greater expansion of ECM in BMSC_SAM/P6+miR-21_ and accumulation of transcripts considered as late markers of osteogenesis. The changeable expression profile of Alpl during osteogenic differentiation was noted previously by other authors [54, 55].

Simultaneously, miR-21 upregulation negatively affected *Trap*, *Ctsk* and *Nfatc-1* expression, thus, rescues osteoporotic BMSC_SAM/P6_ from their osteoclastic-like nature. TRAP has been shown to be a critical cytochemical marker of osteoclasts and its high level in patients’ serum corresponds with bone resorption process in menopausal women [56]. TRAP has been identified as a critical regulator of skeleton development and bone mineralization, collagen synthesis and degradation, production of dendritic cells, as well as macrophages recruitment [15]. Conversely, miR-21 did not change significantly the expression of *Pu.1*; however, we noticed its higher level in BMSC_SAM/P6_ compared to BMSC_BALB/c_. Obtained results stay in line with previous studies that illustrated the important role of *Nfatc-1* and *Pu.1* during osteoclast maturation in murine BMSCs and RAW264.7 cell lines. Essentially, the inadequate level of both transcripts may also be the cause of osteoporosis progression [57–59]. Inhibiting osteoclasts activity might be an important strategy for protection of bone resorption, thus, protection against osteoporosis development.

The activity of particular miRNAs regulating gene expression has been identified as a critical phenomenon that might significantly affect bone formation and bone resorption. In this study, we have found that miR-21-5p increases the expression of miR-7a-5p, miR-145-3p and miR-223-3p in BMSC_SAM/P6+miR-21_. Their enhanced expression might be considered as a good prognosis for miR-21 application due to their significant involvement in maintaining bone formation [2, 60–62]. Interestingly, in this study we observed a dual function of miR-21. Its upregulation resulted in a higher expression of transcripts involved in osteoblast formation in BMSC_SAM/P6_. On the other hand, a reduced expression of miR-17-5p and miR-124-3p that are involved in osteoporosis progression has been evidenced [63–65].

In order to verify the therapeutic potential of miR-21-5p, we performed in vivo bilateral cranial defect model using osteoporotic SAM/P6 mice. Since SAM/P6 mice represent the model of senile osteoporosis with low bone mass and reduced BMD index (bone mineral density), verification of clinical effectiveness using this particular model is fully justified. We observed a higher accumulation of calcium and phosphorus within the defect sites in SAM/P6_miR-21_ group two weeks after the procedure. Additionally, the contribution of newly formed tissue was significantly higher in SAM/P6_miR-21_, when compared to SAM/P6_CTRL_. When combined, these results indicate improved osteogenesis in SAM/P6_miR-21,_ resulting in the intensified formation of functional, highly mineralized bone tissue. Obtained results show the osteoinductive properties of miR-21 confirmed on critical-size cranial defect model. In this study, we have proposed the potential mechanism of miR-21 action indicating its potential targets at mRNA level. We believe that the obtained data sheds a promising light on the potential therapeutic application of miR-21-5p in osteoporotic fractures treatment [50, 66].

## Conclusions

**Fig. 7:**
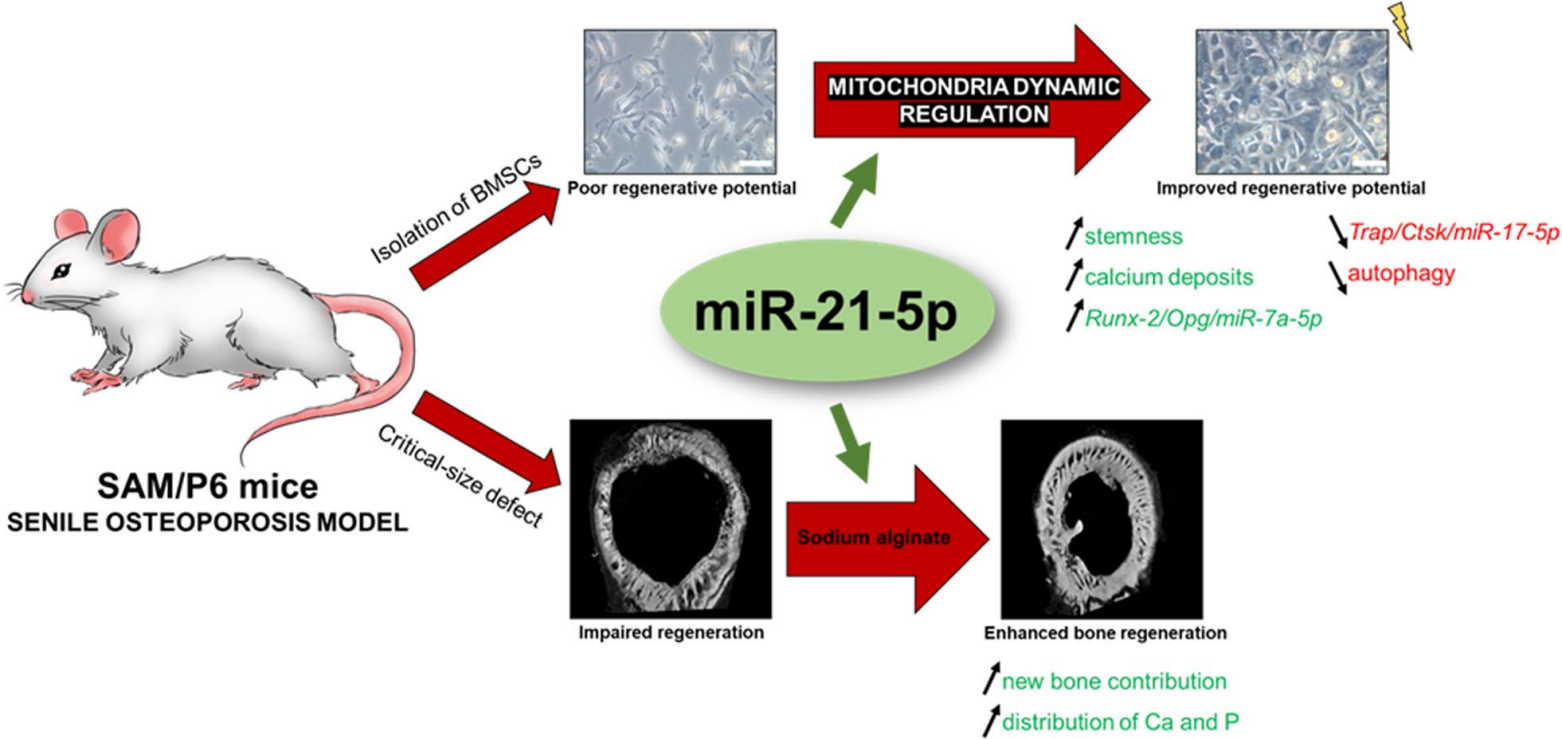
A graphical abstract of performed experiment. Upregulation of miR-21-5p regulates the dynamic of mitochondria network, thus, positively affects the proper mineralization of extracellular matrix in BMSC_SAM/P6_ and accumulation of proteins crucial for bone homeostasis maintenance. MiR-21 decreases the deteriorated autophagy and regulates mitochondrial network dynamic in BMSCs. In vivo study confirms the results obtained during ex vivo experiments, evidenced by enhanced bone regeneration and greater accumulation of Ca and P. All images and illustrations depicted in this figure were prepared by the Authors

Here, we demonstrated for the first time that upregulation of miR-21-5p in BMSCs derived from unique model of senile osteoporotic SAM/P6 mice results in modulation of mitochondria network dynamic, thus rejuvenation of cells’ phenotype and improved bone-forming capability (Fig. 7). We have proven that miR-21 upregulation positively affects the proper mineralization of extracellular matrix in BMSC_SAM/P6_ and accumulation of proteins crucial for bone homeostasis maintenance. Importantly, miR-21 regulates the deteriorated autophagy of BMSCs. Performed in vivo study confirms the results obtained during ex vivo experiments. Obtained results stay in line with our previous papers concerning the effect of miR-21 in murine MC3T3-E1 cell line and BMSCs [20, 28, 50]. However, for the first time the rejuvenating role of miR-21 was identified using BMSCs isolated from senile accelerated SAM/P6 mice, as well as focused on mitochondria metabolism. We believe that obtained results may serve as an important factor during investigations in terms of osteoporosis diagnosis and treatment.

## Supplementary Information

**Additional file 1: Figure S1.** MiR-21 regulates the number and phenotype of mitochondria in BMSCs isolated from osteoporotic SAM/P6 mice. Detailed MicroP analysis of mitochondria number (**A**) as well as mitochondria morphology classified as simple tubes (**B**), branching tubes (**C**) and small globes (**D**). The results are presented as bar graphs. Significant differences between groups are indicated with asterisk: *p < 0.05, **p < 0.01, ***p < 0.001. Non-significant differences are marked as ns.

## Data Availability

The datasets generated during and/or analysed during the current study are available from the corresponding author on reasonable request. The accession numbers presented in Table 1 refer to specific nucleotides and can be found in the official database of National Center for Biotechnology Information (https://www.ncbi.nlm.nih.gov/).

## Abbreviations

ALPL: Alkaline phosphatase
BALB/c: Healthy BALB/c mice strain
BAX: Bcl-2-associated X protein
BCL-2: B-cell lymphoma 2
BMP-2: Bone morphogenetic protein 2
BMSCs: Bone marrow-derived mesenchymal stem/stromal cells
COLL-1: Collagen type 1
CSD: Critical-size cranial defect
CTSK: Cathepsin K
DRP1: Dynamin-related GTPase 1
ECM: Extracellular matrix
FBS: Fetal bovine serum
FIS1: Mitochondrial fission 1 protein
HAp: Hydroxyapatite
HBSS: Hank’s balanced salt solution
IOH: International Osteoporosis Foundation
LAMP2: Lysosome-associated membrane protein 2
MFF: Mitochondrial fission factor
MFN-1: Mitofusin-1
MFN-2: Mitofusin-2
MiD49 & MiD50: Mitochondrial kinetic protein 49 and 50
miRNA/microRNA: Small non-protein-coding RNA
miR-21/miR-21-5p: MicroRNA-21-5p
MMP-9: matrix metalloproteinase 9
MSCs: Mesenchymal stem cells
mTOR: Mammalian target of rapamycin kinase
NFATC-1: Nuclear factor of activated T-cells, cytoplasmic 1
OCL: Osteocalcin
OP: Osteoporosis
OPA1: Optic atrophy protein 1
OPG: Osteoprotegerin
OPN: Osteopontin
PFA: Paraformaldehyde
PU.1: Transcription factor PU.1
PPAR-γ: Peroxisome proliferator- activated receptor gamma
P/S: Penicillin & streptomycin antibiotic
RUNX-2: Runt-related transcription factor 2
RT-qPCR: Reverse transcription followed by a quantitative polymerase chain reaction
SAM/P6: Senescence-accelerated mice prone 6
SEM–EDX: Scanning electron microscope with energy-dispersive X-ray analysis
TRAP: Tartrate-resistant acid phosphatase
WHO: World Health Organization
µ-CT: X-ray computed microtomography
