# Supplementary figures and images for: MiR-21-5p regulates the dynamic of mitochondria network and rejuvenates the senile phenotype of bone marrow stromal cells (BMSCs) isolated from osteoporotic SAM/P6 mice
Source: Stem Cell Res Ther. 2023 Mar 29;14:54. doi: 10.1186/s13287-023-03271-1 (PMC10053106; doi:10.1186/s13287-023-03271-1)

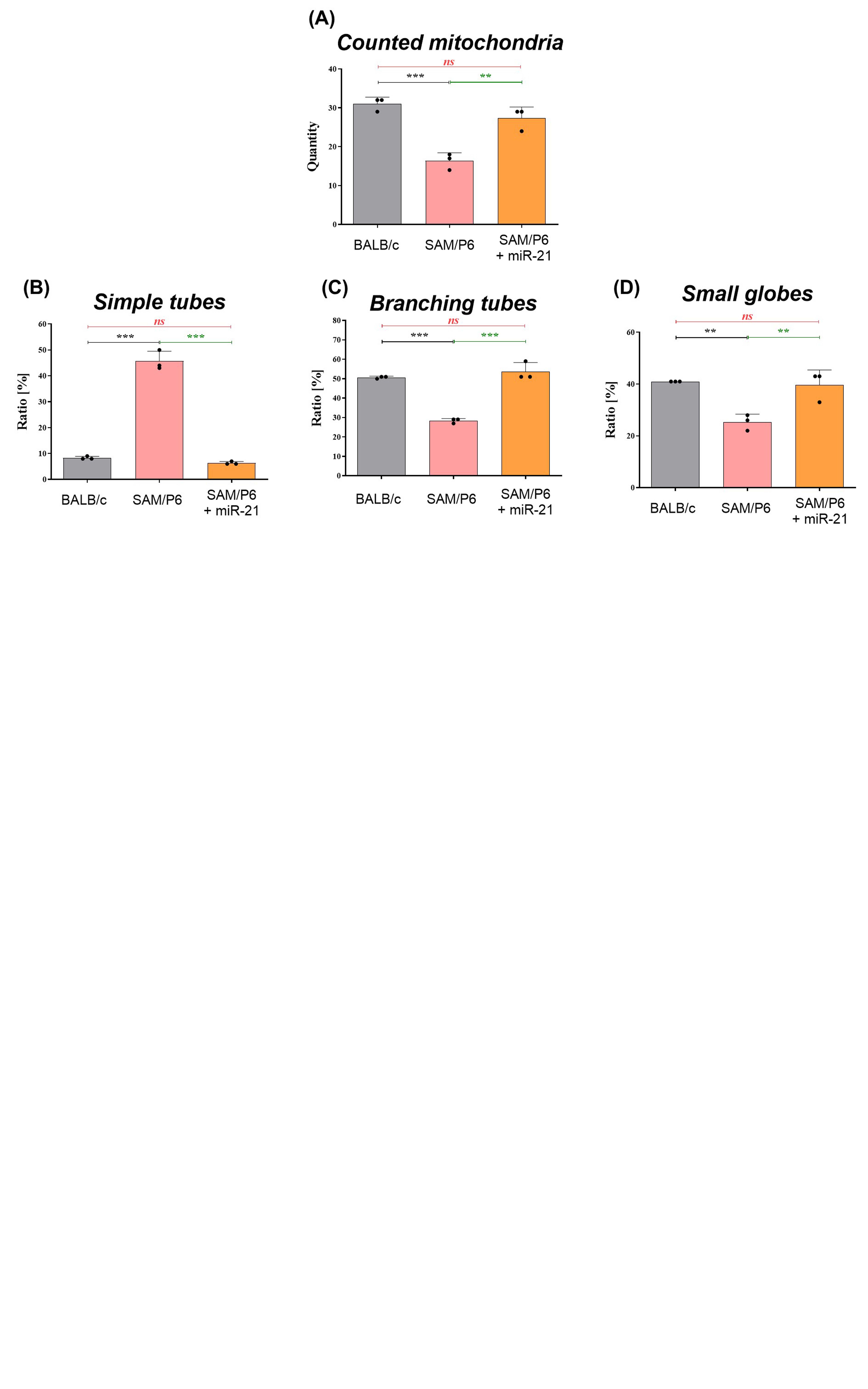

Supplement: Supplementary file 1 — Additional file 1: Figure S1. MiR-21 regulates the number and phenotype of mitochondria in BMSCs isolated from osteoporotic SAM/P6 mice. Detailed MicroP analysis of mitochondria number (A) as well as mitochondria morphology classified as simple tubes (B), branching tubes (C) and small globes (D). The results are presented as bar graphs. Significant differences between groups are indicated with asterisk: *p < 0.05, **p < 0.01, ***p < 0.001. Non-significant differences are marked as ns. [file 13287_2023_3271_MOESM1_ESM.jpg]
